# Strengthening the Bridge Between Academic and the Industry Through the Academia-Industry Collaboration Plan Design Model

**DOI:** 10.3389/fpsyg.2022.875940

**Published:** 2022-06-06

**Authors:** Farah Ahmed, Muhammad Tahir Fattani, Syed Rizwan Ali, Rabia Noor Enam

**Affiliations:** ^1^Department of Software Engineering, Sir Syed University of Engineering and Technology, Karachi, Pakistan; ^2^Business Incubation Center, Bahria University, Karachi, Pakistan; ^3^Computer Engineering Department, Sir Syed University of Engineering and Technology, Karachi, Pakistan

**Keywords:** industry, academia, practitioner, ecosystem, innovation, evolution

## Abstract

The study has been undertaken to integrate two different aspects of the triple helix model: universities and the industry. Special attention has been paid to the prevailing difference between the two, hampering their working as a coherent unit. Integrating the existing knowledge in the study, we proposed the Academia-Industry Collaboration Plan (AICP) design model. The model comprises processes, methods or approaches, and tools. Processes serve as a road map to third parties for establishing collaboration between academia and the industry. It has all the essential process models and a series of steps that help minimize the organizational complexity of the collaboration process between academia and the industry. Methods or approaches serve the purpose of implementing those processes effectively. Finally, appropriate tools are selected to integrate possible collaboration improvements that lead to innovation.

## Introduction

Universities serve the industry in two ways. It provides the workforce necessary to run the industry, and two, it furnishes innovative ideas to start new business ventures. This apparent simplistic relationship does not work so simplistically because of the inherent differences between the two. Universities desire to contribute to the theory. On the other hand, the industry is restrained by profitability. Therefore, academia and the industry are analogous to two sides of a river that must flow independently. As far as science and engineering disciplines are concerned, creating linkages between the two sides of the river has the potential to contribute to the betterment of both—the industry and universities.

According to the organization for economic co-operation and development (OECD), the industry conducts around two-thirds of R&D in science and technology studies. The remaining 20% of R&D work is carried out by universities, while 10% is carried out by the government (Organisation for Economic Co-operation and Development [Oecd], 2017). The world’s top-ranking universities attract industry funding for their projects and innovations. The pharmaceutical industry is one of the most prominent investors in universities (Shehatta and Mahmood, 2016). The IT industry has also started investing in academies because it has concluded that it is beneficial to spend on students and academia. Exactly 26,355 universities from all over the world are included in a survey carried out in July 2016 that lists the countries arranged by the number of universities in the top rankings (Shattock, 2017). With this statistical data, it can be concluded that collaboration between the industry and academia is on the rise. Undertaking research in the industry paves the way for easy dissemination of knowledge, as universities have access to real-time data, eliminating the dichotomy arising from the characteristic differences between the two. This study proposes a model for the desired assimilation of the industry and university, leading to more efficient working of the two.

## Literature Review

During the 1990s revolution of information systems, research on the industrial community has initialized the discussion, but the problem remained in top-ranked journals (Steinbach and Knight, 2006). The research article provides relevance, discussion, and a broad scope of the practitioner community and their issues. It also emphasizes that these problems are not to be resolved on a global, systemic, or academic level; instead, they are determined on an individual basis, specified and defined by the researcher. Different researchers made a brief plan for the particular research problem to find and resolve the targeted problem (Fitzgerald, 2003). This manuscript presents the possible research gap and consensus, which have always made it difficult for academia and the industry to walk together. According to the analytical thinking perspective, the breach between the institution and the industry due to inadequate intended results affects the communication process and the comparatively light reporting of essential technology stuff in the university syllabus. We talk about the conception and bring forward some of the key issues to better understand the foundations of the research-practice breach. Aside from recommending that students exchange their views in acquiring surroundings, this paper discusses how to improve shared empathy between students and researchers in order to increase the capacity for effective collaboration while simultaneously raising the prerequisites of engineering. Numerous papers have been published in peer-reviewed technical journals every year. There is no need to publish a more significant number of papers with an industry affiliation. Most of the papers are just systematic reviews in the software engineering discipline. In Pakistani, students are taught high-end programming languages. Nonetheless, they end up using them for repeated non-industry-based applications like matrix multiplication programs or developing web-based library management systems. They know the syntax of the programming languages but not the domains in which such programming languages should be applied. As students graduate and move to the industry, unless the industry gives them proper training in the field (banking, embedded, military applications, etc.), it is hard for an average student to flourish.

While there has been plenty of research conducted in academia, there is still criticism of academic research. Various patterns and frameworks that need to be addressed remain unaddressed in information science, which includes professional/client collaboration and the right of each stakeholder (Birdsall, 2009). Communicating academic research findings to IS professionals: An analysis of problems written by Lang says that research findings often do not directly or immediately relate to IS professionals in the industry. Lang also stresses the communication gap between researchers and practitioners (Lang, 2002). Though realized very early, the inability of students to cope up with the challenges of real world are persisting. It has become imperative to provide students with a rich experience of the industry along with academic training so they can at least avoid the difficulties arising from being ignorant of the development taking place in the industry or at most become more prolific in combining the beneficial aspects of the two. Academia should focus on high-level skills rather than implanting low-level skills in students. Not everything taught can be found in books or in traditional classrooms. Knowledge and skills can be gained through experience. Knowledge, skills, and equipment provide employees with resources to withstand the pressures emerging from challenges associated with the processes of collaboration (Belli, 2021).

The relationship between knowledge and skills sets, and their gear as a 3-stage social fabric network, sheds light on how one-of-a-kind varieties of researchers function in their engagement with affordance and emotional attachment.

It has been observed that students learn most of the topics during their jobs, including testing verification, quality assurance, project management, ethics and professionalism, technical writing, and leadership skills (Khan et al., 2021a,d, e). Such topics should be included in their academic journey. To motivate the industry to collaborate, we must also encourage academic institutions to do so for mutual benefits. Researchers must develop the curriculum to address the industry’s major problems, considering how things will help both sides. It is necessary to include fundamental industrial practices in academia to help students develop their skills.

Moody (2003) talks about the lengthy delay in disseminating research findings into practice, a process in which some results are inevitably lost (Moody, 2003). Students should be encouraged to work on industry-identified problems and do static validation of their thesis/project work. In the Pakistani scenario, others do not have a mandatory 6-month project in the industry except for medical courses.

In Pakistan, people who cannot get campus placements spend 1 or 2 years getting certified in any field before starting the job hunt. At times, certifications are more valuable than degrees themselves; this need to get certificates clearly states one thing: there is an evident lack of industry knowledge in academia. An interesting myth is that functional programming languages are just for academic purposes, but Erlang, a Prolog-based functional programming language, has been used in Ericson for ages. In universities, people study software testing techniques theoretically, but they do not do much about learning software testing tools, which are used predominantly in the industry for software testing.

There are plenty of training institutions that teach industry-specific skills. Yet, they are generally out of reach financially for students. Industrial research is not made publicly available until it fulfills its business interests. This study has started discussing this topic on a social media platform, bringing out an interesting point. For the companies, institution interaction can only provide intangible benefits that cannot go beyond a slot in campus placement, and they can directly train students during internships (governmental and educational institutions). For any other interaction, companies cannot get returns in terms of money. One way to resolve this is to encourage institutions to hire industry experts as their faculties. Considering that the software industry and software engineering academia are the prominent communities with meager collaborative efforts and few joint projects compared to the size of these communities, researchers and practitioners are less motivated to participate in association with each other. Driven in the opposite direction by their varied objectives, the collaboration between the industry and universities has been limited. It is hard to develop a connection between academia and the industry until issues such as research relevance, training commitment, problem resolution in the real world, communication gap, contractual and privacy concerns are explicitly addressed (Garousi et al., 2017).

The success of students’ projects in collaboration with the industry depends on an ecosystem that involves academia, industry liaison, clients, students, and faculty. Students create products in association with the industry or for the industry and then work as entrepreneurs. Understanding the emergence of new research subjects suggested by university students that may lead to innovation or value creation for the industry requires a combination of imagination and entrepreneurship (Hansson and Mønsted, 2008). Students apply academic analysis to practical work (Hillon et al., 2012). It is a composite task to provide practitioners with knowledge of or abstractions from research and to translate research into practice; therefore, a new discipline must be introduced to bridge the gap between research and practice. Translational development renders research findings reliable, practical results that bridge the gap between academia and industry (Norman, 2010). Planning a collaboration process is imperative; defining a time constraint for long-term and short-term relationships between academia and the industry will eventually aid in understanding the nature of projects that both researchers and practitioners pursue. Time view applies to industry-academic joint projects that determine when the research is estimated to be practiced (Runeson and Minör, 2014). The transformation of knowledge into practice is time-consuming. It takes approximately 4–5 years for applied research to be fully implemented in the industry; the rate of transformation is even slower for basic research. It has been observed that governmental and educational institutions are more visible than research institutes (Ahmed et al., 2019; Belli and Gonzalo-Penela, 2020; Khan et al., 2021b,c).

It is based on a model introduced by Connor et al. (2009) that industry needs are based on five success factors facilitated by research results: needing orientation, industry goal alignment, deployment impact, industry benefit, and innovation. It focuses on research actions like management engagement, network access, collaborator match, communication ability, and continuity (Sandberg et al., 2011). Success factors can be drawn from the previous collaborations formed by the industry and academia (Wohlin et al., 2011). Short- and long-term collaborative efforts specify collaborations. An alliance or partnership between participants can be official or casual. This may vary from partnership equity, agreements, research-based projects, and copyrights to capital flows, publications, and meetings in workshops, focus groups, and seminars (Guimón, 2013). For this study, “collaboration propensity” is based on the likelihood of an individual researcher collaborating at a specific moment in time and in relation to current research interests (Birnholtz, 2007). Education has to adjust itself to the industry and job market changes, but this process requires a consolidated representation of fields and teaching programs (Venant et al., 2015). Working with universities on research projects requires learning to work across organizational boundaries. Also, they can build the capacity to cooperate with participants managing within a different structural motivation system (Bruneel et al., 2010). Universities are boosting their connections with the industry in order to play a massive role in the innovation ecosystem due to the increased significance of the triple helix model. While this connection leads to more knowledge generation and economic prosperity, it also has an effect on university norms (Dooley and Kirk, 2007).

Many chambers of commerce, education ministries, and commerce ministries lack cooperation, resulting in a purposeless and unproductive curriculum that fails to fulfill trade and industry requirements. Another reason for failing to understand and prepare technical and commerce colleges for public and private sector universities to meet the latest trends and market requirements is the absence of infrastructural linkage (Soharwardi, 2011). The industry that develops its processes around work has an inherent penchant for keeping things simple. On the other hand, universities working around semesters work to cover the elongated time. Because one is compelled by the paucity of time and the other swollen by the abundance of the same commodity. The collaboration between the two—the industry and university—is possible if universities can simplify their work for the industry (Michael, 2013). An ideal collaborative situation can be achieved where technological transfer from the industry to academia makes new collaboration possible. Research has new findings from previous investigations, as well as the emergence of new companies where technology finds its way to better cooperation between research development (Fujisue, 1998).

## Academia and Industry Collaboration Plan

As indicated earlier, it has become increasingly clear that bridging the gap between academia and the industry is essential, but even more critical is bridging the gap between requirements engineering in the literature and incorporating those requirements into practices more efficiently. Therefore, identification of the problem domain is needed, which includes the following:

• Use old processes and techniques when researchers have already introduced new methods.
• On the contrary, the theoretically defined process may not be relevant in the industrial field when implemented.
• The processes studied by students are applied in the industry, but their application may vary according to requirements.
• There is a disconnect between academic researchers and industrial researchers.
• A student, in many cases, has no idea about how to market themselves outside of academia.
• Availability of resources provided to researchers and practitioners.
• The industry is reluctant to implement new ideas, whereas academia is reluctant to adopt new teaching ideas.
• The trust deficit between the two partners is low in the long run.

Both partners should realize that technological development is impossible without new ideas. It has become necessary to build a long-term relationship between primary stakeholders. Therefore, we are underlining significant features in the solution domain, which may be the inception of a collaboration plan between academia and the industry.

• Theoretical standards should be practically performed in industrial environments.
• An internship program should be initiated.
• Project managers for industrial personnel with explicit knowledge will conduct lectures to disseminate knowledge sharing with academia.
• Academia shares new research with the industry and prepares reports on new process models that benefit their partners.
• Industrial relevance is important when teaching students.
• Sharing resources is needed between the industry and academia. Students and employees should have access to online resources so that they can get references from current research, reports, and books.
• Collaboration should not be restricted to intra-country, but exchanges between students and employees should be done internationally.
• The formation of committees has become necessary for monitoring and expanding this collaboration.

These are the few things that must be done before entering the next phase of AICP. It is essential to understand the need for interaction between academia and the industry, and these collaborative interactions need to be based on mutual benefits.

### Academia-Industry Collaboration Plan’s New Approach in Connection With the Triple Helix Model

Innovation requires a breeding ground, which is furnished by an innovative environment. The triple helix model (THM) is an approach that can be employed to pursue the creation of an innovation-friendly environment. This model connects the field of practice (industry) with regulatory authority (government) to a knowledge repository (universities) to facilitate innovation. The three interacting components—industry, government, and universities—compensate for the shortcomings of each other. The industry desirous of the solution seeks a knowledge source for ideas that are duly provided by universities linked to the industry (Dooley and Kirk, 2007). THM also connects the industry with the government. Being directly linked to the government, the industry can mitigate the hassle of bureaucratic processes that are characteristic of governmental organizations.

Universities can benefit from this interaction with the industry in many ways. First, academicians need to have a practical problem to which they can apply their knowledge. When universities are connected to the industry, they are flooded with practical issues demanding solutions. Additionally, being linked to the industry, universities also have an authentic source of data. Second, universities can also revamp their curriculum in light of their interaction with the industry. By doing so, universities increase the employability of their graduates. Finally, the link between universities and the industry connects the advancement of knowledge with the advancement of practice. When problems are approached in a collaborative manner, the chance of a new startup increases exponentially.

**FIGURE 1:**
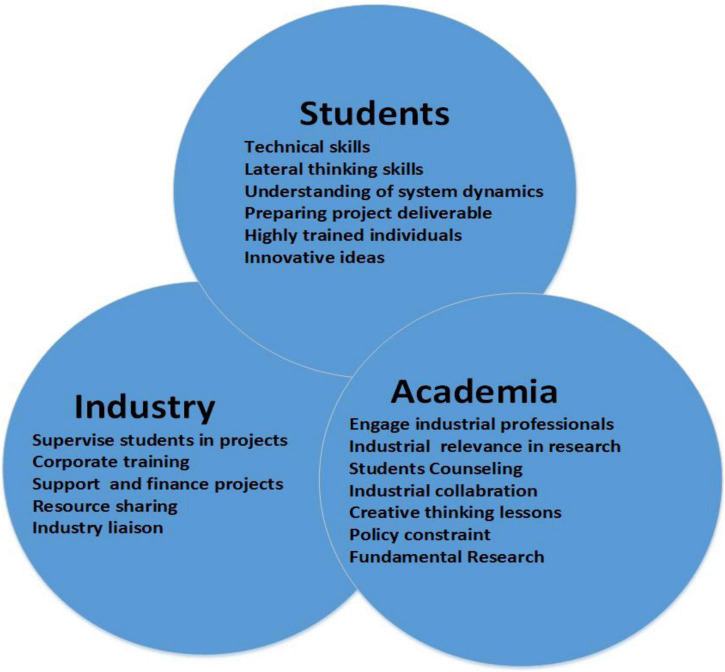
Role of students, industry, and academia (Iyer, 2014).

The AICP design model furnishes students with an opportunity to be connected to the industry. So, once they are graduated, the search for a job turns out to be simple. Graduating students, using their established connections, easily find job opportunities. A vast pool of opportunities for students who can implement their ideas with the help of industries will be created. Academia suggests that the sector improve/replace the current procedures with more productive ones. On the other hand, enterprises can send trained professionals to teach/guide the students. They can also give students activities related to their courses and help the industrialists improve their functionality as shown in Figure 1.

#### Initial Process

• Initiation
  (a) The need to pitch the importance of collaboration between the industry and academia.
  (b) How can this collaboration bring about productive changes in both sectors?

#### Expansion and Specifications

• Academia
  (a) Industrial professionals share their expertise and work behavior with students, giving them a comprehensive outlook on professional work.
  (b) Help students find relevancy between theoretical and practical approaches.
  (c) They can do theoretical research work and suggest its practical implications in the industrial environment.
• Industry
  (a) They can provide activities that help strengthen students’ innovative capabilities, giving them constructive ideas for application in the industry for effective functioning.
  (b) Ideas can be made possible if they are backed by expertise.
  (c) Rather than sending their employees on expensive training, their professionalism can be improved by their personal learning experience by communicating and expressing their ideas with students and getting feedback from their perspective.
  (d) They can cut short their recruitment process by hiring the students they are already working in collaboration with.
• Implementation process
  (a) A mutual consensus is required from both sides of the industry and academia.
  (b) It is essential to have the industrialists help guide academia.
• Monitoring and review
  (a) Most importantly, constant monitoring is needed, as is a weekly-to-monthly review process for proper functioning.
  (b) When a problem occurs that needs immediate action, a proactive approach should mostly be mainly acquired.
• Feedback
  (a) It is most important to get feedback on the collaborative approach as a whole, either through weekly or monthly seminars, to resolve problems that arise.

### How Can This Collaboration Bring About Productive Changes in Both Sectors?

(a) Industrial professionals share their expertise and work behavior with students, giving them a comprehensive understanding of professional working.
(b) Help students find relevancy between theoretical and practical approaches.
(c) They can conduct academic research and suggest its practical implications in an industrial environment.
(d) Ideas can be made possible if they are backed by expertise.
(e) Instead of sending their employees on expensive training, industries can increase the professionalism of their employees through their personal learning experiences by communicating and expressing their ideas with students and getting feedback from their perspectives.
(f) They can cut short their recruitment process by hiring the students they are already working in collaboration with.
(g) Both sides can quickly do a mutual consensus.
(h) When both partners work together and adhere to the same standards, problems are faced and resolved more effectively.

## Framework Activity for Increasing Communication Between the Industry and the Institution

It is important to create a framework activity that leads to developing the task sets and work products to improve communication between academia and the industry, resulting in an extended relationship that allows for the mass introduction and sharing of ideas between them. It is evident from the software engineering perspective that such activities define guidelines and set standards for initiating a task. The framework activities for the test set of communications extending the relationship between academia and the industry for collaborative work in the field of research and implementation of that research would be as follows:

• Planning
  (1) Information gathering
  (2) Adapting a model for communication and information sharing
  (3) Scheduling workshops and seminars for students and industrialists
• Analysis and specifications
  (1) Promoting creative activities/small tasks to judge students and to find out whether they are suitable
  (2) Identifying relevant industries for students’ internships or visits
  (3) List down the appropriate institution for industrial collaboration
  (4) Eradicating unnecessary behaviors
  (5) Meetings
  (6) Suggesting new ideas from both the industry and universities
• Design and review
  (1) Acceptance criteria
  (2) Proposal writing
  (3) Verification and validation
  (4) Team design
  (5) Student’s group formation and reviews
  (6) Acceptance of new ideas
• Implementation of feedback rework
  (1) Interactive sessions and focus groups
  (2) Information sharing
  (3) Working projects
  (4) Internships
  (5) New theoretical knowledge transfer in industries
  (6) Implementation of the latest ideas in collaboration

**FIGURE 2:**
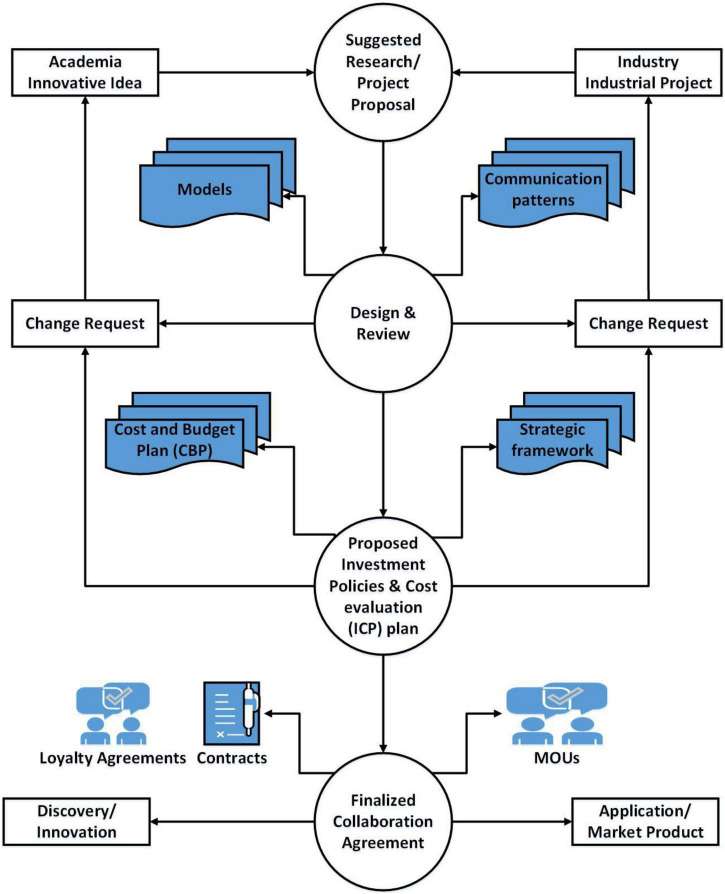
Academia industry collaboration (AIC) engineering process architecture.

Keeping in mind the current scenario analysis and collaboration standards for academia and the industry, a basic strategic planning structure that incorporates two-way communication is needed. Figure 2 shows open-minded communication between academia and industry, with information sharing and knowledge-sharing abilities. Such a system will provide a complete and dynamic collaboration while strengthening relationships. Bridging the divide between universities and practitioners is an important task performed, and its negligence would lead to project failure. Among the few salient features are identifying the stakeholders and prioritizing them, which should be addressed thoroughly before initiating collaborative efforts. The resilient stakeholders to uplift the dynamics of research and the implementation of ideas into inventions come from academia and industries that help implement new ideas into reality. There is a massive gap between academics and practitioners, and industries ignore when engineers and scientists are in a production state.

On the other hand, academia neglects the fact that these students follow market trends. There must be industrial concentration in educational institutions, whereas educational institutions must know the requirements of industries. There might be several barriers to adopting an outside idea if only the idea were given without the insight and proper understanding of all its dynamics. The essential factor noted against adapting the idea is that it is hard to find relevance in academic or independent study research material and implementation of practices. The independent study researches a significantly less popular among the practitioners, consumers, and organizations because the researcher hardly focuses on implementing their recommendations; the idea encoded by the researcher most of the time never gets decoded by practitioners (Obrist et al., 2012). Practitioners’ lack of interest in independent study research for students’ project ideas is a myth that research is a medium of expert solutions. The reality of this myth is that it lets students disclose their thoughts and ideas in a more generic way, which will help them not only eradicate the problem but also help them enhance their knowledge.

Research can be relevant and fruitful for practitioners if it shows strength in both of the discussions; one is to provide solutions, or at the very least a way toward a solution, and the other is to determine how long theories be discriminated with time and need. The adaptability of an idea is also an aspect to think about. On the other hand, asking for immediate solutions and results from researchers in a short period is not appropriate. Practitioners and academics alike must devote time to it. Academics must focus on material relevance; work products and projects are necessary for industrial and academic collaboration. We should cultivate a culture that enables us to understand that we are searching for an idea that may not be the result itself. Indeed, communication in the proliferation of products is very important.

These are only a few factors that might trigger a situation of divergence associated with the weakened relationship between academia and the industry. That may be derived from effective teaching, peer learning, and knowledge sharing between the universities and industries (Connor et al., 2009). One must understand that research cannot be transformed into a result unless the proper two-way model is identified and adapted as per the requirements, which vary with the situation. Universities providing opportunities for research and development must give them an edge in their fields. A diversity of ways to incorporate new ideas must be created, and standards should be created for the growth of research and development.

## Academia Industry Collaboration Engineering Process Model

Academia industry collaboration (AIC) is complex because of the diverse needs of various stakeholders and the engineering process involved. The AIC engineering process model can expand the scope of initiation and identify the plan for instituting a suggestion or proposal for transforming inputs into derived requirements. Suggested plans or proposed research are converted into the desired output and innovated problem-solving product or application. The AIC engineering process model explains the architecture implied by the higher-lever to lowest-level requirements proposed by academia or the industry.

It also expresses the plan for anticipated changes and the creation of work products in each process. The AIC engineering process provides a clear system perspective and guidelines for selecting or developing practical methods and tools. This model organizes the results, requests for changes, and processes engineering to ensure the contingency of effective collaboration between academia and the industry, as shown in Figure 2.

## Academia Industry Collaboration Design Model

**FIGURE 3:**
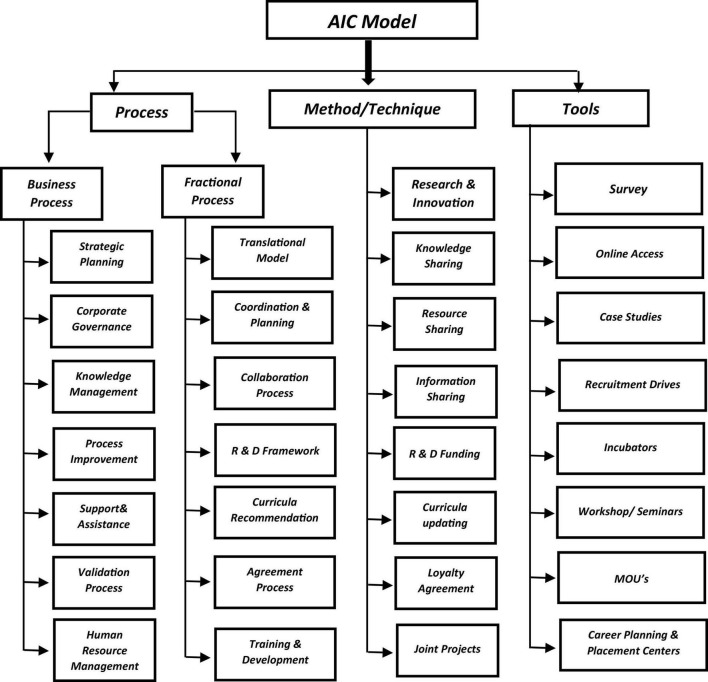
Academia industry collaboration (AIC) design model-A concise plan to initiate and execute.

The AIC design model comprises processes, methods or approaches, and tools, as shown in Figure 3. The processes will serve as a road map to third parties for establishing collaboration between academia and the industry. It has all the essential process models and a series of steps that will help minimize the organizational complexity of the collaboration process between academia and the industry.

Each process indicated in Figure 3 is based on mutual goals and relies on mutual trust to move forward with greater relevance and a concise plan to initiate and execute. Each process impacts the decision-making methodology that will lead to substantial agreements and disagreements between the industry and academia.

A list of a few essential processes has been proposed, which can work as a guideline throughout the collaboration process. Once collaborating parties have decided on the process in which they can work together most effectively, they can then select the methods or approaches that can serve the purpose of implementing those processes effectively. Finally, by using the right tools, we can integrate possible collaboration improvements that lead to innovation.

### Communication Strategies for Academia-Industry Collaboration Plan

**FIGURE 4:**
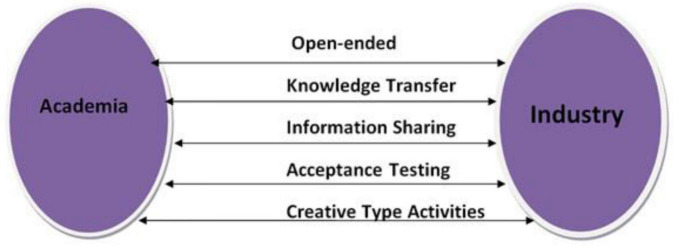
Strategic planning structure for communication between academia and industries.

Forming a flexible and dynamic communication structure between the two potential stakeholders is imperative. The relationship is built on solid communication and the determination to create a self-motivated research environment, as shown in Figure 4. Therefore, a memorandum of understanding to bridge the gap between academia and the industry must be signed.

Approaches to strengthen the bond will include the following:

• Before commencing the task, ways of communication must be identified and acceptance criteria established.
• There must be meetings and collaborations between institutions and organizations.
• Industrial trends should be followed, and topics related to them must be taught.
• Industrial trends and learning strategies full students must be adopted.
• Industrial collaboration with faculty and members (ICT-industrial collaboration team).
• Progressive meetings with students, ICT, and industries should be conducted and recorded.
• A web portal must be created to establish connections and information sharing between researchers and practitioners.

On the other hand, academia and the industry should collectively work on unearthing students’ analytical thinking skills. A plan should be proposed for creative students that encourages the development of new ideas as a collaborative effort. Research funding and incubators will also enhance the essence of research and development.

### Points to Strengthen

There is a weak link between the industry and academia that is not enough. The following points could be considered to strengthen the link:

• Research involving industries can directly be undertaken.
• Universities are making academic research papers available at lower prices.
• Universities are encouraging students to take up industry-identified projects.
• Universities should make it mandatory to have an industrial guide for their projects and thesis.
  Education is a service, but the industry is not, and it is meant for business. University funds can be given for giving training to students.
• The need for adding domain-specific subjects to the curriculum.
• The government can pass a rule mandating industries to train a certain number of university students in a year and requiring companies to be in touch with at least one university.
• Frequent guest lectures may be held, inviting people from the industry.
• It is bringing in industry experts to assist in developing the curriculum.
• Incorporating one person from academia in the research with industry-sponsored research.
• Compulsory 1-year industrial training in addition to studies (like in medical studies).

### Current Trends in the Proposed Policy and Plan

Educational policies are present, but there are obstacles to policy implementation. Often, most organizations pay attention to the auditing process. It becomes necessary to convert policymaking plans into action plans, or it can be managed every year to access improvement and review current trends of the proposed policy and plan. The industry and academia work in entirely different dimensions. Their efforts are disintegrated, and resources are deteriorating because the industry focuses mainly on market-oriented research for food profits, while academia does impact-based research just for the purpose of publishing in journals and presenting new findings at conferences.

**FIGURE 5:**
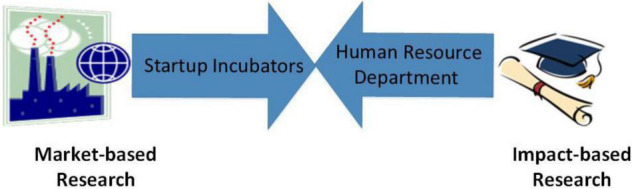
Industry and academia integrated approach.

Due to a lack of organizational and institutional structure in research and development, there is no proper allocation of resources and findings to bridge this gap. Policies must be constituted, and previously approved procedures can be regenerated, reviewed, and reproduced to integrate and implement the collaborative environment. At level 0, this void can be filled by setting up a human resource department that will help bring the industry and academia closer to working in collaboration. The next level must be upgraded by allocating resources and funding for establishing the startup incubators depicted in Figure 5.

There are many other concerns to address, including trust issues between stakeholders. Third-party involvement, such as that of the HR department or career planning and placement bureaus, plays a significant role in ensuring that there shall be no trust issues between academic researchers and the industry that will sabotage the collaboration’s long-term impact. Therefore, loyalty agreements should be signed, and policies should be made and protected in this regard. Academic researchers sign agreements for incentives and protection of the idea, whereas it is equally beneficial for innovation and productivity of industrial products.

They are promoting an unconventional yet innovative culture to help channel our resources, leading to a sustainable long-term relationship between the industry and academia. Formal regulatory bodies in collaboration with the private and public sectors improve knowledge sharing and creative thinking. By striking a balance between structural thinking and rational thinking, a culture of research and innovation can easily be adopted by institutions and industry.

Level 0, the establishment of a human resource department, and Level 1, the establishment of startup incubators, fuel the process of collaboration to the next level, where the innovation committee is an interdisciplinary correspondence that encourages teamwork and entrepreneurship. HR sustains a healthy relationship between academia and the industry and defines an integrated framework that supports academia in funding and marketing the result of research (product of a researcher). On the contrary, incubation directly impacts the industry’s growth and brings market-oriented products that are not duplicated.

Repetitive errors emerge by adapting conventional structures for managing and maintaining collaboration between the industry and academia. Traditional structural needs to trends that are inactive and non-functional. The industry’s thinking pattern develops solution-based market products here as academia follows a conventional theoretical plan for every possible piece of research, formulating more questions. Both follow a different approach and trend. By combing their interests in one particular thing, we can create a long-term merger and collaboration among those entities. Research and development through innovation and creative thinking will provide market solutions and new technological improvements that interest both industry and academia. New innovative ideas and creative thinking can be achieved by conducting university brainstorming sessions to channel students’ ability to think outside the box. The industry is motivated and takes an unconventional approach by allocating a quota of resources for innovation-based market products.

## Conclusion

The industries neither want the sand nor the baked finished pot, but the processed soil can take any shape they wish. The handshaking between industrial research and academic research shall lead to the betterment of the studies and a better economy; however, there are opportunities to produce good (related to school and learning) research that can help the industry. First, it is extremely important to understand industry needs. It can be very hard if the industry does not know what it wants, as is often the case, and does not understand the research process. Even when industry members hold undergraduate degrees, there may not be a described/explained understanding of how research is produced; there may be a need to identify research gaps and ask the industry if they are interested to identify issues that are sometimes ahead of what the industry perceives to be important. This manuscript has offered a prominent level of a summary of comparatively new rules and amendments for improving engineering level students, as well as how students should take industrial training, advice, and suggestions from industry experts, which should also be taken to deliver lectures on time. Students should be encouraged to participate in different workshops, seminars, and training sessions alongside their studies. This manuscript also provided some directions for the future growth of the syllabus and finding out schemes that can be applied to the curriculum.

## Author Contributions

FA: literature review, AICP model, and methodology. MT: discussion, AICP model, and design modeling. SA: abstract, introduction, conclusion, and AICP with triple helix model. RE: literature review and results. All authors contributed to the article and approved the submitted version.

## Conflict of Interest

The authors declare that the research was conducted in the absence of any commercial or financial relationships that could be construed as a potential conflict of interest.

## Publisher’s Note

All claims expressed in this article are solely those of the authors and do not necessarily represent those of their affiliated organizations, or those of the publisher, the editors and the reviewers. Any product that may be evaluated in this article, or claim that may be made by its manufacturer, is not guaranteed or endorsed by the publisher.
